# Effects of Moderate-Intensity Continuous Training and High-Intensity Interval Training on Testicular Oxidative Stress, Apoptosis and m6A Methylation in Obese Male Mice

**DOI:** 10.3390/antiox11101874

**Published:** 2022-09-21

**Authors:** Zujie Xu, Ying Qin, Binbin Lv, Zhenjun Tian, Bing Zhang

**Affiliations:** 1 Division of Sports Science and Physical Education, Tsinghua University, Beijing 100084, China; 2 Institute of Sports Biology, College of Physical Education, Shaanxi Normal University, Xi’an 710119, China

**Keywords:** obesity, MICT, HIIT, testicular, oxidative stress, apoptosis, N6-methyladenosine (m6A) methylation

## Abstract

Exercise is an effective way to improve reproductive function in obese males. Oxidative stress and apoptosis are important pathological factors of obesity-related male infertility. Accumulating studies have demonstrated that N6-methyladenosine (m6A) methylation is associated with obesity and testicular reproductive function. Our study aimed to investigate and compare the effect of 8 weeks of moderate-intensity continuous training (MICT) and high-intensity interval training (HIIT) on testicular oxidative stress, apoptosis and m6A methylation in obese male mice. Male C57BL/6 mice were randomly allocated into the four groups: normal diet (ND) group, high-fat diet (HFD) group, high-fat diet with moderate-intensity continuous training (HFD-MICT) group and high-fat diet with high-intensity interval training (HFD-HIIT) group. Mice in the HFD-MICT and HFD-HIIT groups were subjected to 8 weeks of MICT or HIIT treadmill protocols after 12 weeks of HFD feeding. We found that MICT and HIIT increased the protein expression of Nrf2, HO-1 and NQO-1 in the testes of obese mice, and HIIT increased it more than MICT. The Bax/Bcl-2 ratio, Cleaved Caspase-3 protein expression and TUNEL-positive cells were consistently up-regulated in the testes of obese mice, but MICT and HIIT restrained these HFD-induced effects. In addition, HFDs increased m6A levels and the gene expression of METTL3, YTHDF2 and FTO in the testes, but these effects were reversed by MICT and HIIT. However, HIIT was more effective than MICT in reducing m6A methylation in the testes of obese mice. These results demonstrate that both MICT and HIIT protected against HFD-induced oxidative stress, apoptosis and m6A methylation in testicular tissues; as a result, testicular morphological and functional impairment improved. In particular, HIIT was more beneficial than MICT in increasing the mRNA expression of steroidogenic enzymes and testicular antioxidant capacity and decreasing m6A methylation in the testes of HFD-fed mice.

## 1. Introduction

Obesity is known to be a major risk factor for testicular dysfunction [1,2]. Growing evidence has shown that obesity impairs reproductive functions and causes disturbance of the endocrine axis in males, which is characterized by reduced testicular steroidogenesis and low levels of serum follicle-stimulating hormone (FSH), luteinizing hormone (LH) and testosterone [3,4,5]. Several previous studies have suggested that oxidative stress and apoptosis-mediated testicular damage are important causes of reduced reproductive function in obese males [6,7,8]. However, the mechanisms of obesity-induced testicular dysfunction remain unknown.

N6-methyladenosine (m6A) is the most ubiquitous internal RNA modification that can regulate mRNA degradation, splicing, translation and stability [9]. m6A RNA modification is regulated by m6A-related genes, including m6A methyltransferases (methyltransferase-like 3 (METTL3), METTL14 and WT1-associated protein (WTAP), termed as “writers”), RNA demethylases (alpha-ketoglutarate-dependent dioxygenase (FTO) and AlkB homolog 5 (ALKBH5), termed as “erasers”) and RNA binding proteins (YTH N6-methyladenosine RNA binding protein 1 (YTHDF1), YTHDF2, YTHDF3, YTH domain containing 1 (YTHDC1) and YTHDC2, termed as “readers”) [10,11]. Recent studies have reported that m6A RNA modification can regulate mammalian spermatogenesis and reproductive function [12,13]. In addition, it has been reported that m6A modification is related to obesity, oxidative stress and apoptosis [14,15]. However, the role of m6A modification in high-fat diet (HFD)-induced testicular injury remains unclear.

The antioxidant and anti-apoptotic effects of regular physical activity in chronic diseases have been widely confirmed [16,17]. Accumulating studies have demonstrated that moderate exercise can inhibit HFD-induced testicular oxidative stress and apoptosis, and improve sperm quality and reproductive function [18,19]. However, most studies on obesity-induced testicular injury have focused on the effects of moderate-intensity continuous training (MICT) and there are limited studies about the effects of high-intensity interval training (HIIT) and its protective mechanisms. Therefore, due to the importance of selecting the correct and effective exercise intensity and type to improve testis function, the present study aimed to investigate and compare the effect of 8 weeks of MICT and HIIT on testicular oxidative stress, apoptosis and m6A methylation in obese male mice.

## 2. Materials and Methods

### 2.1. Animals

Male C57BL/6 mice (3- to 4-week-old) were obtained from the Laboratory Animal Research Center of Tsinghua University. Mice were housed in the Laboratory Animal Research Center of Tsinghua University with a 12:12-h light–dark cycle and controlled temperature (24 ± 2 °C). All experimental procedures were approved by the Institutional Animal Care and Use Committee of Tsinghua University (Animal Welfare Assurance no. F16-00228; A5061-01).

After 1 week of adaptation, animals were randomly allocated into the four groups: normal diet (ND) group, high-fat diet (HFD) group, high-fat diet with moderate-intensity continuous training (HFD-MICT) group and high-fat diet with high-intensity interval training (HFD-HIIT) group, with *n* = 15 for each group. The nutritional composition of ND and HFD was reported in a previous article [20].

### 2.2. Exercise Protocol

**Figure 1:**
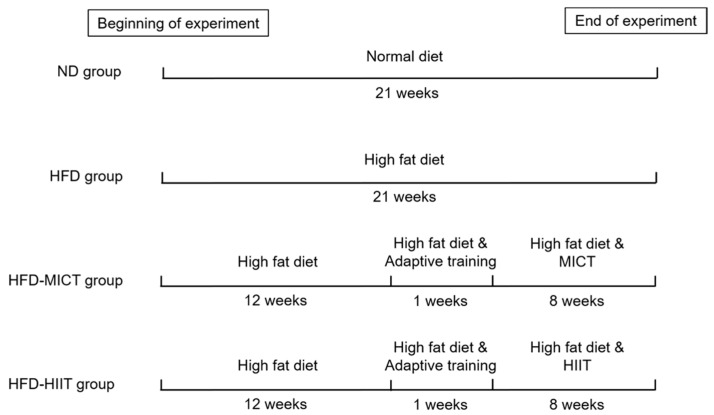
Schematic diagram of experimental design. ND: normal diet; HFD: high-fat diet; MICT: moderate-intensity continuous training; HIIT: high-intensity interval training.

Mice in the HFD-MICT and HFD-HIIT groups were subjected to 8 weeks of treadmill exercise after 12 weeks of HFD feeding. The exercise protocols were slightly modified according to previous studies [21,22]. First, the mice in the exercise groups underwent adaptive training on treadmills for 1 week (5 m/min, 10 min/day, 0°). During the formal training, both groups warmed up for 10 min at a speed of 5 m/min. The HFD-MICT group performed the continuous endurance running for 35 min, and the speed was 14–17.5 m/min. In the HFD-HIIT group, 2 min of moderate-intensity (14–17.5 m/min) treadmill exercise was alternated with 1 min of high-intensity (24–27.5 m/min) treadmill exercise until the running distance was equal to that of the MICT. Training sessions were performed during the morning, five times per week, for 8 weeks. Specific training programs for MICT and HIIT are detailed in the Supplementary Information, Tables S1 and S2. At the end of the 8-week training, mice were sacrificed after fasting for 12 h, and serum and testicles were collected for further analysis. The body weights and testicular weights of the mice were also recorded. The experimental design is shown in Figure 1.

### 2.3. Serum Hormone Measurement

Serum levels of FSH, LH, testosterone and estradiol (E2) were analyzed using mouse enzyme-linked immunosorbent assay (ELISA) kits (Meimian Biotech, Yancheng, China) according to the manufacturer’s instructions. The intra-assay coefficients of variation (CV) of serum FSH, LH, testosterone and E2 were 6.9%, 5.7%, 7.4% and 6.5%, respectively. Optical density (OD) at 450 nm was performed using a microplate meter. Then, the serum sex hormone levels were calculated according to the standard curve.

### 2.4. Kit Assays

Testicular oxidative stress levels were determined by measuring malondialdehyde (MDA), hydrogen peroxide (H_2_O_2_), total superoxide dismutase (T-SOD), catalase (CAT), glutathione peroxidase (GSH-Px) and glutathione (GSH) activity using commercial kits (Nanjing Jiancheng Bioengineering Institute, Nanjing, China) as described previously [23]. The intra-assay CVs of MDA, H_2_O_2_, T-SOD, CAT, GSH-Px and GSH were 4.2%, 3.6%, 4.5%, 5.3%, 5.8% and 4.8%, respectively. All detecting operations were performed following the kits manufacturer’s instructions.

### 2.5. Semen Analysis

The epididymis was carefully separated from testicular fat. The sperm were gradually pressed out from the epididymis and the sperm number was counted with a haemocytometer. Sperm motility was assessed blindly under a light microscope by classifying 200 sperms per animal as either progressive motile, nonprogressive motile or immotile. Sperm motility was expressed as a percentage of motile sperm to total sperm.

### 2.6. Histological Analysis

Mouse testicular tissues were fixed in 4% paraformaldehyde for 24 h and embedded in paraffin. Tissue sections of 5 μm were stained with hematoxylin and eosin (H&E) for histological analysis of testes.

### 2.7. TUNEL Staining

Apoptosis of testicular tissue was assessed by terminal deoxynucleotidyl transferase (TdT) dUTP nick-end labeling (TUNEL) staining. In brief, 5 μm testicular tissue sections were incubated with TUNEL solution (Beyotime, Nanjing, China) in the dark at 37 °C for 1 h. 4′,6-diamidino-2-phenylindole (DAPI) was used to identify the nuclei of testicular cells. The sections were observed under a fluorescence microscope (Nikon, Tokyo, Japan) and the green TUNEL-positive apoptotic cells were counted in randomly selected fields.

### 2.8. m6A RNA Methylation Quantification

m6A levels in testicular tissue were assessed using an m6A RNA quantification kit (Epigentek, Farmingdale, NY, USA), as described previously [20]. Briefly, 200 ng total RNA was incubated with capture antibody at room temperature for 1 h, then m6A antibody was captured and washed. The intra-assay CV of m6A levels was 3.9%. The absorbance was measured at 450 nm and the m6A RNA methylation levels of testicular tissue were calculated from the absorbance value.

### 2.9. Quantitative Real-Time PCR (RT-PCR)

Total RNA was isolated from testicular tissues by using the Trizol reagent and was reverse-transcribed by a PrimeScript™ RT reagent kit (TaKaRa, Tokyo, Japan). RT-PCR was conducted by using SYBR Green PCR Master Mix (Beyotime, Nanjing, China) in the CFX96 Real-Time PCR System (Bio-Rad, Hercules, CA, USA). The mRNA levels were determined using the 2^−ΔΔCt^ method and glyceraldehyde-3-phosphate dehydrogenase (GAPDH) was used as an internal control. Primary sequences used in this study are shown in the Supplementary Information, Table S3.

### 2.10. Western Blot

The total protein was extracted from testicular tissues using cold RIPA buffer. The protein concentration was evaluated by a BCA Protein Assay kit. Equal amounts of protein samples were separated by Sodium dodecyl sulfate-polyacrylamide gel electrophoresis (SDS-PAGE) and transferred to polyvinylidene fluoride (PVDF) membranes. The membranes were blocked with 5% nonfat dry milk for 1.5 h at room temperature and then incubated with the primary antibodies at 4 °C overnight. The antibodies that we used include: 4-HNE (Abcam, Cambridge, MA, USA), Nrf2 (Santa Cruz Biotechnology, Dallas, TX, USA), HO-1 (Abcam, Cambridge, MA, USA), NQO1 (Abcam, Cambridge, MA, USA), Bax (Cell Signaling Technology, Beverly, MA, USA), Bcl-2 (Bioworld, Bloomington, MN, USA), Cleaved Caspase-3 (Cell Signaling Technology, Beverly, MA, USA) and GAPDH (Proteintech, Wuhan, China). The membranes were incubated with the appropriate horseradish-peroxidase (HRP)-conjugated secondary antibodies at room temperature for 1.5 h. Immunopositive bands were visualized by the Image Systems (Bio-Rad, Hercules, CA, USA). The density of immunoblots was calculated by Image Lab software.

### 2.11. Statistical Analysis

All data were obtained from at least three independent experiments and recorded as the mean ± standard error of the mean (SEM) using GraphPad Prism 6. All data in the present study were normally distributed, as determined by the Kolmogorov–Smirnov test. Statistical differences among all groups were analyzed using one-way analysis of variance (ANOVA) followed by post hoc least significant difference (LSD) tests. *p* < 0.05 was considered statistically significant (* *p* < 0.05 and ** *p* < 0.01 are shown in figures).

## 3. Results

### 3.1. MICT and HIIT Improved HFD-Induced Pathological Damage of Testes

**Figure 2:**
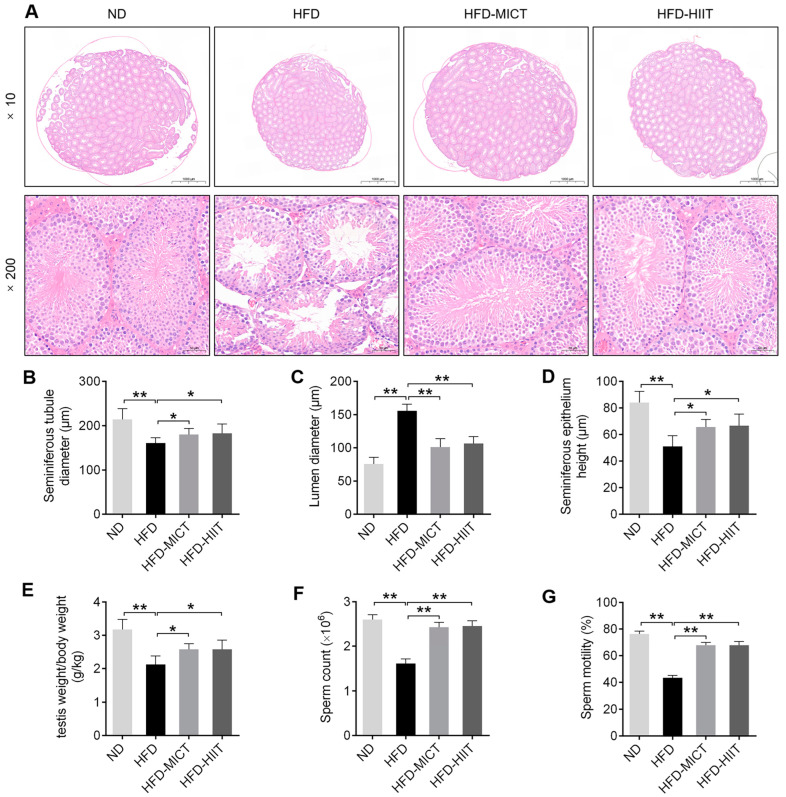
Effects of MICT and HIIT on testicular morphology in obese mice. (**A**) HE staining of mouse testes at 10× (scale bar = 1000 μm) and 200× (scale bar = 50 μm) magnification. Statistical analysis of (**B**) seminiferous tubule diameter, (**C**) lumen diameter, (**D**) seminiferous epithelium height, (**E**) testis weight/body weight ratio, (**F**) sperm count and (**G**) sperm motility. Data are expressed as mean ± SEM. * *p* < 0.05, ** *p* < 0.01.

HFD treated mice exhibited atrophied testes and vacuolation of the seminiferous tubules, and 8 weeks of MICT and HIIT significantly improved abnormal testicular structure (Figure 2A). Moreover, HFD feeding resulted in increased lumen diameter and decreased seminiferous tubule diameter, seminiferous epithelium height, testis weight/body weight ratio, sperm count and sperm motility, while both MICT and HIIT significantly attenuated these effects, and there were no significant changes among the two exercise protocols (Figure 2B–G).

### 3.2. HIIT, but Not MICT, Attenuated HFD-Induced Abnormal Serum Sex Hormone Levels

**Figure 3:**
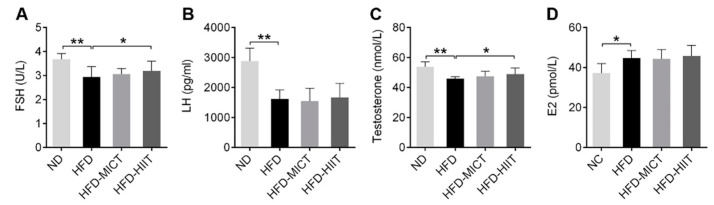
Effects of MICT and HIIT on serum levels of FSH, LH, testosterone and E2 in obese mice. (**A**) FSH, (**B**) LH, (**C**) testosterone and (**D**) E2 were detected in serum. Data are expressed as mean ± SEM. * *p* < 0.05, ** *p* < 0.01.

HFD feeding caused significant decreases in serum FSH, LH and testosterone levels, and significant increases in E2 levels compared with the ND group (Figure 3A–D). After 8 weeks of HIIT, serum FSH and testosterone levels showed a significant recovery, but these changes did not occur in the HFD-MICT group (Figure 3A,C). MICT and HIIT had no effect on serum E2 and LH levels (Figure 3B,D).

### 3.3. MICT and HIIT Increased the Steroidogenic Enzymes’ mRNA Expression after an HFD-Induced Decrease

**Figure 4:**
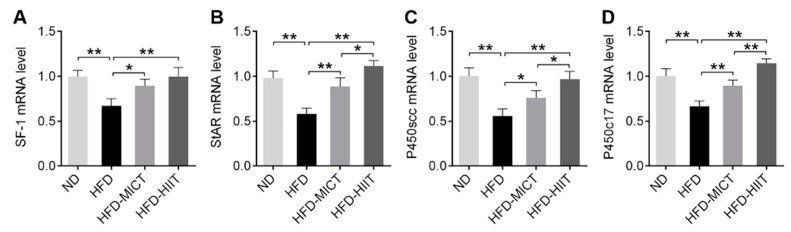
Effects of MICT and HIIT on mRNA expression of steroidogenic enzymes in the testes of obese mice. The gene expression of (**A**) SF-1, (**B**) StAR, (**C**) P450scc and (**D**) P450c17 in mouse testes were detected by RT-PCR. Data are expressed as mean ± SEM. * *p* < 0.05, ** *p* < 0.01.

We analyzed the mRNA expression of steroidogenic enzymes, including SF-1, StAR, P450scc and P450c17. We found that the mRNA levels of SF-1, StAR, P450scc and P450c17 in testes were decreased by HFDs. MICT and HIIT treatment significantly reversed these effects (Figure 4A–D). Moreover, the mRNA levels of StAR, P450scc and P450c17 in the HFD-HIIT group were higher than those in the HFD-MICT group (Figure 4B–D).

### 3.4. MICT and HIIT Suppressed Testicular Oxidative Stress Induced by HFD

**Figure 5:**
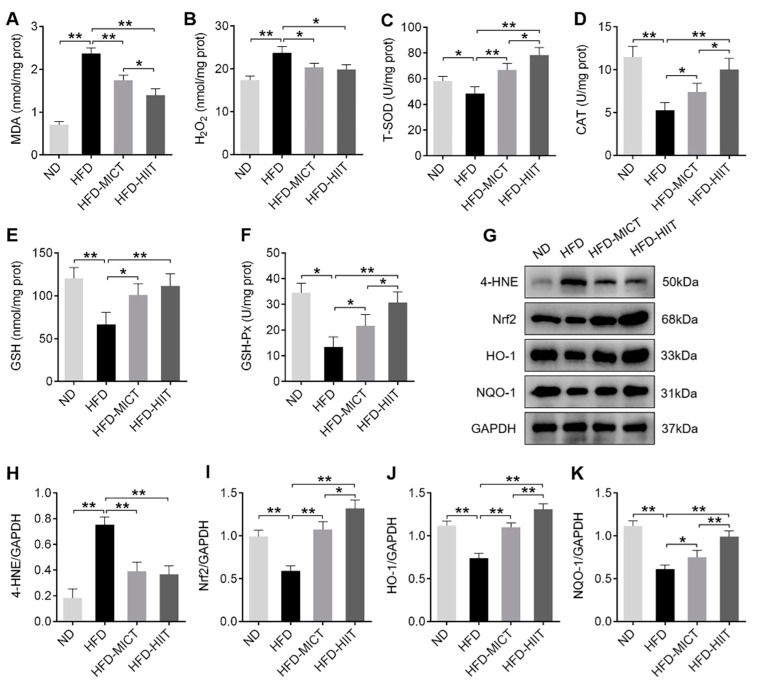
Effects of MICT and HIIT on testicular oxidative stress in obese mice. The activities of (**A**) MDA, (**B**) H_2_O_2_, (**C**) T-SOD, (**D**) CAT, (**E**) GSH and (**F**) GSH-Px in mouse testes. (**G**–**K**) Protein levels of 4-HNE, Nrf2, HO-1 and NQO-1 were detected by Western blot. Data are expressed as mean ± SEM. * *p* < 0.05, ** *p* < 0.01.

The activities of MDA and H_2_O_2_ in testes were significantly up-regulated, and the activities of T-SOD, CAT, GSH and GSH-Px were significantly down-regulated by HFD, but these effects were obviously reversed by MICT and HIIT treatment. Additionally, the MDA content in the HFD-HIIT group was significantly lower than that in the HFD-MICT group, while the activities of T-SOD, CAT and GSH-Px in the HFD-HIIT group were significantly higher than those in the HFD-MICT group (Figure 5A–F). Western blot analysis showed that the decreased Nrf2, HO-1 and NQO-1 protein expression were up-regulated and 4-HNE protein expression was down-regulated in the testes of obese mice after MICT and HIIT treatment. Moreover, the protein expression of Nrf2, HO-1 and NQO-1 in the HFD-HIIT group were significantly higher than those in the HFD-MICT group (Figure 5G–K). These results suggested that HIIT was more advantageous in enhancing testicular antioxidant capacity in obese mice.

### 3.5. MICT and HIIT Decreased HFD-Induced Testicular Apoptosis

**Figure 6:**
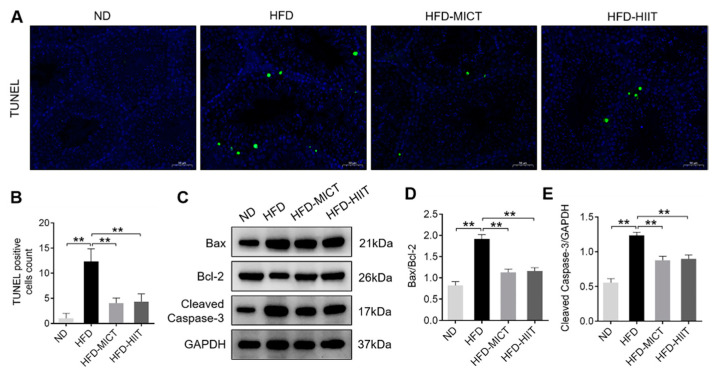
Effects of MICT and HIIT on testicular apoptosis in obese mice. (**A**) TUNEL staining and (**B**) quantitative analysis of mouse testes. The positive apoptotic particles are in green and the nuclei are in blue. (**C**–**E**) Protein levels of of Bax, Bcl-2 and Cleaved Caspase-3 were detected by Western blot. Data are expressed as mean ± SEM. ** *p* < 0.01.

TUNEL staining revealed that HFD-induced elevations in testicular positive apoptotic particles were inhibited by MICT and HIIT (Figure 6A,B). Western blot analysis showed that HFDs resulted in significant up-regulation of Bax/Bcl-2 ratio and Cleaved Caspase-3 protein expression, which were attenuated by MICT and HIIT (Figure 6C–E). These findings indicated that MICT and HIIT decrease testicular apoptosis in HFD-fed mice.

### 3.6. MICT and HIIT Reduced m6A Methylation in the Testicular Tissue of Obese Mice

**Figure 7:**
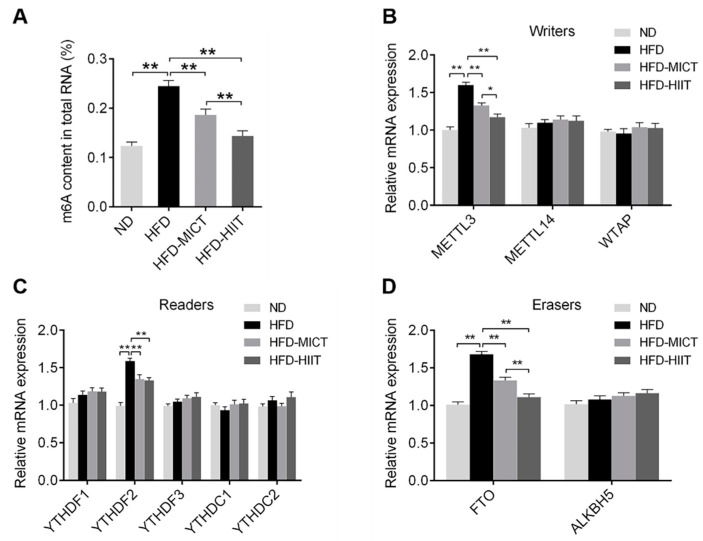
Effects of MICT and HIIT on testicular m6A methylation in obese mice. (**A**) The m6A content in mouse testes. The gene expression of (**B**) “writers” (METTL3, METTL14 and WTAP), (**C**) “readers” (YTHDF1, YTHDF2, YTHDF3, YTHDC1 and YTHDC2) and (**D**) “erasers” (FTO and ALKBH5) in mouse testes were detected by RT-PCR. Data are expressed as mean ± SEM. * *p* < 0.05, ** *p* < 0.01.

To assess the effect of MICT or HIIT on testicular m6A methylation, we measured m6A level and m6A-associated genes. HFD significantly increased m6A level and the gene expression of METTL3, YTHDF2 and FTO, but these effects were reversed by MICT and HIIT (Figure 7A–D). Moreover, the m6A content and the gene expression of METTL3 and FTO in the HFD-HIIT group were significantly lower than those in the HFD-MICT group (Figure 7B,D). These results demonstrated that HIIT was more effective than MICT in reducing m6A methylation in the testes of obese mice.

## 4. Discussion

Obesity is closely related to male reproductive dysfunction [24]. Oxidative stress and apoptosis are important pathological factors of obesity-related male infertility [25,26]. Exercise training is considered to be an effective way to improve reproductive function in obese males [27,28]. In this study, we found that 8 weeks of MICT and HIIT protected against HFD-induced oxidative stress, apoptosis and m6A methylation in testicular tissues; as a result, testicular morphological and functional impairment improved. In particular, HIIT was more efficient than MICT in increasing the mRNA expression of steroidogenic enzymes and testicular antioxidant capacity and decreasing m6A methylation in the testes of obese mice. To the best of our knowledge, this is the first study to report the effects of exercise on m6A methylation, and the first study to compare MICT and HIIT as potential treatments to improve testicular injury in obese mice.

Increasing evidence suggests that long-term HFD feeding induces sex hormone disorders and testicular reproductive dysfunction [6,29]. It has been reported that 8 weeks of swimming exercise with moderate load can significantly increase testosterone synthesis and sperm quality, and ameliorate obesity-related reproductive dysfunction in HFD-fed mice [18]. As expected, our study found that MICT and HIIT improved HFD-induced pathological damage of testes by decreasing lumen diameter and increasing seminiferous tubule diameter and seminiferous epithelium height. In addition, MICT and HIIT could also increase the mRNA expression of steroidogenic enzymes in testes, including SF-1, StAR, P450scc and P450c17. The protective effects of HIIT were better than MICT. There is a literature report that moderate-intensity swimming training can significantly increase serum LH level and decrease E2 level in obese mice [18]. In this study, we found that neither MICT nor HIIT had significant effects on serum LH and E2, which may be due to the different exercise modes and amounts. Of note, 8 weeks of HIIT, but not MICT, significantly increased serum FSH and testosterone levels. These findings suggested that MICT and HIIT could ameliorate obesity-related reproductive dysfunction from multiple levels such as improving testicular structure, sex hormone and steroidogenic enzymes expression.

Oxidative stress plays an essential role in obesity-induced reproductive disorders [8,30]. Obesity can lead to systemic oxidative stress, which induces ROS accumulation [31]. When the levels of MDA and 4-HNE of the lipid peroxidation byproducts were higher than the activity of antioxidant enzymes, testes were vulnerable to oxidative damage [32]. A previous study reported that lifelong running inhibited oxidative stress in the testes of aging mice by reducing the levels of 4-HNE and H_2_O_2_ [33]. Another study showed that 8 weeks of swimming training significantly reduced oxidative stress levels and increased antioxidant capacity in the testes of obese mice [18]. In line with these findings, our study found that MICT and HIIT decreased the activities of MDA and H_2_O_2_ but restored the activities of T-SOD, CAT, GSH and GSH-Px in the testes of HFD-fed mice. Interestingly, we found that the antioxidant capacity of HIIT was better than MICT, which implied that higher training intensities induced greater changes in the antioxidant defense. Studies have shown that the Nrf2 antioxidant pathway takes vital functions in cellular antioxidant defense [34,35]. Nrf2 knockout mice resulted in increased testicular oxidative stress and decreased antioxidant capacity and reproductive capacity, suggesting that Nrf2 plays a key role in preventing oxidative disruption of spermatogenesis [36]. In this study, we reported that MICT and HIIT increased the protein expression of Nrf2, HO-1 and NQO-1 in the testes of obese mice, and HIIT increased more than MICT. Therefore, based on the current results, we speculated that higher antioxidant capacity may be closely related to higher protein expression of Nrf2, HO-1 and NQO-1 in the testes of HFD-HIIT group mice.

Oxidative stress has been reported to induce testicular apoptosis [37]. Studies have shown that testicular apoptosis increased in the testes of HFD-fed mice [38,39]. Another study showed that aerobic exercise inhibited testicular apoptosis in obese rats by reducing Bax/Bcl-2 ratio and TUNEL-positive cells [40]. Similarly, our study found that the Bax/Bcl-2 ratio, Cleaved Caspase-3 protein expression and TUNEL-positive cells were consistently up-regulated in the testes of obese mice, while MICT and HIIT reversed these HFD-induced effects. These findings confirmed that exercise induced testicular protective effects on inhibiting apoptosis.

Accumulating studies have demonstrated that m6A methylation is specifically associated with obesity and testicular reproductive function [12,14]. Our previous study reported that HFD resulted in increased m6A methylation levels and METTL3 expression in the myocardium of mice, while decreasing FTO expression and inducing myocardial apoptosis [20]. In addition, m6A methylation and its regulatory factors are abnormally expressed in testicular diseases, leading to testicular oxidative damage, apoptosis and reproductive dysfunction [13,15]. It was reported that m6A methylation levels and the expression of METTL3 and METTL14 were up-regulated in semen samples of patients with asthenozoospermia [41]. Diphthalate (DEHP) is a common environmental endocrine disruptor, which increases the expression of FTO and YTHDC2 to up-regulate testicular m6A levels, inducing testicular injury and reproductive disorders [15]. A recent study showed that m6A RNA methylation plays an essential role in the modulation of testosterone synthesis in Leydig cells [42]. In the present study, HFD increased m6A level and the gene expression of METTL3, YTHDF2 and FTO in the testes, but these effects were reversed by MICT and HIIT. Interestingly, we found that HIIT was more effective than MICT in reducing m6A methylation in the testes of obese mice, suggesting that m6A levels may be modulated by exercise intensity.

In conclusion, our study suggests that 8 weeks of MICT and HIIT are effective therapeutic strategies for reducing testicular oxidative stress, apoptosis and m6A methylation in HFD-fed mice. Our study may contribute to the design of better nonpharmacological intervention protocols for obesity-related male infertility. There are some limitations in the current study. As a descriptive study, our experiment lacked knockout animals to further explore the exact molecular mechanisms by which exercise ameliorates testicular injury in obese mice. Additionally, further studies are needed to understand the molecular mechanisms leading to the higher benefits of HIIT over MICT, increasing antioxidant capacity and decreasing m6A methylation in the testes of obese mice.

## 5. Conclusions

Eight weeks of MICT and HIIT protected against HFD-induced oxidative stress, apoptosis and m6A methylation in testicular tissues; as a result, testicular morphological and functional impairment improved. In particular, HIIT was more beneficial than MICT in increasing the mRNA expression of steroidogenic enzymes and testicular antioxidant capacity and decreasing m6A methylation in the testes of HFD-fed mice.

## Data Availability

Data is contained within the article and supplementary material.

## Supplementary Materials

The following supporting information can be downloaded at: https://www.mdpi.com/article/10.3390/antiox11101874/s1, Table S1: MICT protocol over 8 weeks; Table S2: HIIT protocol over 8 weeks; Table S3: Primer sequences used for RT-PCR.

## References

[1] Oliveira P.F., Sousa M., Silva B.M., Monteiro M.P., Alves M.G. (2017). Obesity, energy balance and spermatogenesis. Reproduction.

[2] Liu Y., Ding Z. (2017). Obesity, a serious etiologic factor for male subfertility in modern society. Reproduction.

[3] Pasquali R., Patton L., Gambineri A. (2007). Obesity and infertility. Curr. Opin. Endocrinol. Diabetes Obes..

[4] Paasch U., Grunewald S., Kratzsch J., Glander H.J. (2010). Obesity and age affect male fertility potential. Fertil. Steril..

[5] Hammoud A.O., Gibson M., Peterson C.M., Meikle A.W., Carrell D.T. (2008). Impact of male obesity on infertility: A critical review of the current literature. Fertil. Steril..

[6] Rato L., Alves M.G., Cavaco J.E., Oliveira P.F. (2014). High-energy diets: A threat for male fertility?. Obes. Rev..

[7] Leisegang K., Sengupta P., Agarwal A., Henkel R. (2021). Obesity and male infertility: Mechanisms and management. Andrologia.

[8] Barbagallo F., Condorelli R.A., Mongioi L.M., Cannarella R., Cimino L., Magagnini M.C., Crafa A., La Vignera S., Calogero A.E. (2021). Molecular Mechanisms Underlying the Relationship between Obesity and Male Infertility. Metabolites.

[9] Xu Z., Lv B., Qin Y., Zhang B. (2022). Emerging Roles and Mechanism of m6A Methylation in Cardiometabolic Diseases. Cells.

[10] Zaccara S., Ries R.J., Jaffrey S.R. (2019). Reading, writing and erasing mRNA methylation. Nat. Rev. Mol. Cell Biol..

[11] Shi H., Wei J., He C. (2019). Where, When, and How: Context-Dependent Functions of RNA Methylation Writers, Readers, and Erasers. Mol. Cell..

[12] Cai Z., Niu Y., Li H. (2021). RNA N6-methyladenosine modification, spermatogenesis, and human male infertility. Mol. Hum. Reprod..

[13] Liu S., Lao Y., Wang Y., Li R., Fang X., Gao X., Dong Z. (2021). Role of RNA N6-Methyladenosine Modification in Male Infertility and Genital System Tumors. Front. Cell Dev. Biol..

[14] Sun M., Zhang X. (2021). Epigenetic regulation of N6-methyladenosine modifications in obesity. J. Diabetes Investig..

[15] Zhao T.X., Wang J.K., Shen L.J., Long C.L., Liu B., Wei Y., Han L.D., Wei Y.X., Wu S.D., Wei G.H. (2020). Increased m6A RNA modification is related to the inhibition of the Nrf2-mediated antioxidant response in di-(2-ethylhexyl) phthalate-induced prepubertal testicular injury. Environ. Pollut..

[16] Gomez-Cabrera M.C., Domenech E., Vina J. (2008). Moderate exercise is an antioxidant: Upregulation of antioxidant genes by training. Free Radic. Biol. Med..

[17] Pahlavani H.A. (2022). Exercise-induced signaling pathways to counteracting cardiac apoptotic processes. Front. Cell Dev. Biol..

[18] Yi X., Tang D., Cao S., Li T., Gao H., Ma T., Yao T., Li J., Chang B. (2020). Effect of Different Exercise Loads on Testicular Oxidative Stress and Reproductive Function in Obese Male Mice. Oxidative Med. Cell. Longev..

[19] You T., Disanzo B.L., Arsenis N.C. (2013). Aerobic exercise training attenuates obesity-related hypogonadism in male rats. Med. Sci. Sports Exerc..

[20] Xu Z., Qin Y., Lv B., Tian Z., Zhang B. (2022). Intermittent Fasting Improves High-Fat Diet-Induced Obesity Cardiomyopathy via Alleviating Lipid Deposition and Apoptosis and Decreasing m6A Methylation in the Heart. Nutrients.

[21] Liu Y., Dong G., Zhao X., Huang Z., Li P., Zhang H. (2020). Post-exercise Effects and Long-Term Training Adaptations of Hormone Sensitive Lipase Lipolysis Induced by High-Intensity Interval Training in Adipose Tissue of Mice. Front. Physiol..

[22] Xiong Y., Chen Y., Liu Y., Zhang B. (2020). Moderate-Intensity Continuous Training Improves FGF21 and KLB Expression in Obese Mice. Biochemistry.

[23] Wu F., Li Z., Cai M., Xi Y., Xu Z., Zhang Z., Li H., Zhu W., Tian Z. (2020). Aerobic exercise alleviates oxidative stress-induced apoptosis in kidneys of myocardial infarction mice by inhibiting ALCAT1 and activating FNDC5/Irisin signaling pathway. Free Radic. Biol. Med..

[24] Du Plessis S.S., Cabler S., McAlister D.A., Sabanegh E., Agarwal A. (2010). The effect of obesity on sperm disorders and male infertility. Nat. Rev. Urol..

[25] Suleiman J.B., Mohamed M., Abu Bakar A.B., Zakaria Z., Othman Z.A., Nna V.U. (2022). Therapeutic Effects of Bee Bread on Obesity-Induced Testicular-Derived Oxidative Stress, Inflammation, and Apoptosis in High-Fat Diet Obese Rat Model. Antioxidants.

[26] Heydari H., Ghiasi R., Ghaderpour S., Keyhanmanesh R. (2021). The Mechanisms Involved in Obesity-Induced Male Infertility. Curr. Diabetes Rev..

[27] Palmer N.O., Bakos H.W., Owens J.A., Setchell B.P., Lane M. (2012). Diet and exercise in an obese mouse fed a high-fat diet improve metabolic health and reverse perturbed sperm function. Am. J. Physiol. Endocrinol. Metab..

[28] Di Luigi L., Romanelli F., Sgro P., Lenzi A. (2012). Andrological aspects of physical exercise and sport medicine. Endocrine.

[29] Ding N., Zhang X., Zhang X.D., Jing J., Liu S.S., Mu Y.P., Peng L.L., Yan Y.J., Xiao G.M., Bi X.Y. (2020). Impairment of spermatogenesis and sperm motility by the high-fat diet-induced dysbiosis of gut microbes. Gut.

[30] Abbasihormozi S.H., Babapour V., Kouhkan A., Niasari Naslji A., Afraz K., Zolfaghary Z., Shahverdi A.H. (2019). Stress Hormone and Oxidative Stress Biomarkers Link Obesity and Diabetes with Reduced Fertility Potential. Cell J..

[31] Fernandez-Sanchez A., Madrigal-Santillan E., Bautista M., Esquivel-Soto J., Morales-Gonzalez A., Esquivel-Chirino C., Durante-Montiel I., Sanchez-Rivera G., Valadez-Vega C., Morales-Gonzalez J.A. (2011). Inflammation, oxidative stress, and obesity. Int. J. Mol. Sci..

[32] Aitken R.J., Roman S.D. (2008). Antioxidant systems and oxidative stress in the testes. Oxid. Med. Cell Longev..

[33] Chigurupati S., Son T.G., Hyun D.H., Lathia J.D., Mughal M.R., Savell J., Li S.C., Nagaraju G.P., Chan S.L., Arumugam T.V. (2008). Lifelong running reduces oxidative stress and degenerative changes in the testes of mice. J. Endocrinol..

[34] Rotimi D.E., Ojo O.A., Olaolu T.D., Adeyemi O.S. (2022). Exploring Nrf2 as a therapeutic target in testicular dysfunction. Cell Tissue Res..

[35] Hybertson B.M., Gao B., Bose S.K., McCord J.M. (2011). Oxidative stress in health and disease: The therapeutic potential of Nrf2 activation. Mol. Aspects Med..

[36] Nakamura B.N., Lawson G., Chan J.Y., Banuelos J., Cortes M.M., Hoang Y.D., Ortiz L., Rau B.A., Luderer U. (2010). Knockout of the transcription factor NRF2 disrupts spermatogenesis in an age-dependent manner. Free Radic. Biol. Med..

[37] Shan W., Lu S., Ou B., Feng J., Wang Z., Li H., Lu X., Ma Y. (2021). PACAP ameliorates the fertility of obese mice through PAC1/PKA/ERK/Nrf2 signal axis. J. Endocrinol..

[38] Ghosh S., Mukherjee S. (2018). Testicular germ cell apoptosis and sperm defects in mice upon long-term high fat diet feeding. J. Cell Physiol..

[39] Bunay J., Gallardo L.M., Torres-Fuentes J.L., Aguirre-Arias M.V., Orellana R., Sepulveda N., Moreno R.D. (2021). A decrease of docosahexaenoic acid in testes of mice fed a high-fat diet is associated with impaired sperm acrosome reaction and fertility. Asian J. Androl..

[40] Li N.C., Wei X.X., Hu Y.L., Hou X., Xu H. (2018). Aerobic exercise blocks interleukin-6 levels and germ cell apoptosis in obese rats. Andrologia.

[41] Yang Y., Huang W., Huang J.T., Shen F., Xiong J., Yuan E.F., Qin S.S., Zhang M., Feng Y.Q., Yuan B.F. (2016). Increased N6-methyladenosine in Human Sperm RNA as a Risk Factor for Asthenozoospermia. Sci. Rep..

[42] Chen Y., Wang J., Xu D., Xiang Z., Ding J., Yang X., Li D., Han X. (2021). m(6)A mRNA methylation regulates testosterone synthesis through modulating autophagy in Leydig cells. Autophagy.

