# Supplementary material for: Effects of Moderate-Intensity Continuous Training and High-Intensity Interval Training on Testicular Oxidative Stress, Apoptosis and m6A Methylation in Obese Male Mice
Source: Antioxidants (Basel). 2022 Sep 21;11(10):1874. doi: 10.3390/antiox11101874 (PMC9598593; doi:10.3390/antiox11101874)
Supplement: Supplementary file 1 [file antioxidants-11-01874-s001.zip › antioxidants-1855104-supplementary.pdf]

Supplemental Information

Table S1. MICT protocol over 8 weeks.

	Warm up 45～50% VO_{2max}		Moderate intensity 70～75% VO_{2max}		Total distance (m)	Total time (min)
	Pace (m/min)	Time (min)	Pace (m/min)	Time (min)		
1w	5	10	14	35	540	45
2w			14.5		557.5	
3w			15		575	
4w			15.5		592.5	
5w			16		610	
6w			16.5		627.5	
7w			17		645	
8w			17.5		662.5	

Table S2. HIIT protocol over 8 weeks.

	Warm up 45～50% VO_{2max}		Moderate intensity 70～75% VO_{2max}		High intensity 95～100% VO_{2max}		Total distance (m)	Total time (min)
	Pace (m/min)	Time (min)	Pace (m/min)	Time (min)	Pace (m/min)	Time (min)		
1w	5	10	14	2×10	24	1×9	540	38.6
2w			14.5		24.5		557.5	38.8
3w			15		25		575	39
4w			15.5		25.5		592.5	39.1
5w			16		26		610	39.2
6w			16.5		26.5		627.5	39.3
7w			17		27		645	39.4
8w			17.5		27.5		662.5	39.5

Table S3. Primer sequences used for RT-PCR.

Genes	Primer Sequences	
	Forward (5′–3′)	Reverse (5′–3′)
SF-1	TTCTGAGAGCCCGCTAGCCACT	CGTCCGCTGAACGGAAGGAGAA
StAR	AAAGCCAGCAGGAGAACGGGGA	GCCTCCATGCGGTCCACAAGTT
P450scc	CTGCCTGGGATGTGATTTTCA	GTAATGTTGGCCTGGATGTTCT
P450c17	GATCGGTTTATGCCTGAGCG	TCCGAAGGGCAAATAACTGG
METTL3	CTGGGCACTTGGATTTAAGGAA	GTATCCCATCCAGTTGGTTTC
METTL14	CTGAGAGTGCGGATAGCATTG	GAGCAGATGTATCATAGGAAGCC
WTAP	TAGACCCAGCGATCAACTTGT	CCTGTTTGGCTATCAGGCGTA
FTO	TTCATGCTGGATGACCTCAATG	GCCAACTGACAGCGTTCTAAG
ALKBH5	GCATACGGCCTCAGGACATTA	TTCCAATCGCGGTGCATCTAA
YTHDF1	ACAGTTACCCCTCGATGAGTG	GGTAGTGAGATACGGGATGGGA
YTHDF2	GAGCAGAGACCAAAAGGTCAAG	CTGTGGGCTCAAGTAAGGTTC
YTHDF3	GATCAGCCTATGCCATATCTGAC	CCCCTGGTTGACTAAAAACACC

YTHDC1	GGAAGCACCCAGTGTATAGGA	GGAAGCACCCAGTGTATAGGA
YTHDC2	GAAGATCGCCGTCAACATCG	GCTCTTTCCGTACTGGTCAAA
GAPDH	GCAAGGACACTGAGCAAGA	GGATGGAAATTGTGAGGGAG
